# Supplementary material for: Discrimination of pollen of New Zealand mānuka (Leptospermum scoparium agg.) and kānuka (Kunzea spp.) (Myrtaceae)
Source: PLoS One. 2022 Jun 3;17(6):e0269361. doi: 10.1371/journal.pone.0269361 (PMC9165797; doi:10.1371/journal.pone.0269361)
Supplement: S1 File — (DOCX) [file pone.0269361.s001.docx]

**S1 Notes. Comparison of pollen dimensions of male and bisexual flowers of *Leptospermum scoparium* s.l. and *Kunzea robusta***

**Material and Methods**

This pilot study was based on a small number of samples of male and bisexual flowers collected fresh from plants at two localities in the Tairawhiti (East Cape) region. The samples are in pairs, each pair comprising one sample of several male flowers and one sample of several bisexual flowers from each of three plants of *Leptospermum scoparium* s.l. and three plants of *Kunzea robusta* (Table 1). At each locality, the plants sampled are considered to be a single genetic population.

The specimens were received in 15 ml centrifuge tubes, several flowers per tube, suspended in ethanol. This was topped up with distilled water, and each tube vigorously stirred before decanting half of the suspension into fresh labelled tubes. Each of the sample suspensions was then sieved through a 90 µm sieve cloth to remove non-pollen flower parts, and concentrated by centrifugation at 3000 rpm for 5 minutes. The residual pollen samples were then prepared using Erdtman’s acetolysis method, as described in the main paper. All samples were prepared at the same time. The samples were left in water for 1 hour, then centrifuged, decanted, and microscope slides made up for each using glycerine jelly with safranin stain as mounting medium. Slides are retained in the GNS Science pollen reference collection with NZGS registration numbers.

Measurements of equatorial (E) and polar diameters (P) were made under the highest magnification available on the Zeiss Axio.Imager microscope used, using a 100x oil immersion objective, 1.6x tube factor, and a 10x eyepiece with an eyepiece graticule of 100 divisions. At this magnification, each division is equivalent to 0.625 µm. Measurements were made to the nearest half division. To avoid unconscious bias in the selection of pollen grains for measurement, every grain encountered in suitable orientation in one or two traverses across the middle part of the area under the microscope slide coverslip was measured. This was continued until totals of 30 equatorial diameters and 10 polar diameters were achieved. Folded or damaged pollen grains were ignored. Specimens of pale and thin-walled pollen grains were ignored as suspected immature pollen grains.

**Results**

**Table 1. Pollen dimensions of *L. scoparium* s.l. and *K. robusta* specimens.**

| **Plant** | **Species** | **Collection number (Newstrom-Lloyd)** | **Collection locality** | **Flower sex^a^** | **NZGS#** | **E, n = 30**  **Min (Mean) Max^b^** | **E**  **σ^b^** | **P, n = 10**  **Min (Mean) Max^b^** | **P**  **σ^b^** |
| --- | --- | --- | --- | --- | --- | --- | --- | --- | --- |
| 1 | *L. scoparium* s.l. | 20131122-01 LN | Willi Kaa apiary site, Tikitiki, Tairāwhiti, N Island NZ | M | 4835 | 14.06 (17.06) 18.44 | 0.9 | 10.63 (10.84) 11.25 | 0.26 |
|  |  |  |  | B | 4836 | 15.63 (17.13) 19.38 | 0.73 | 10.63 (10.84) 11.25 | 0.26 |
| 2 | *L. scoparium* s.l. | 20131122-03 LN C | Willi Kaa apiary site, Tikitiki, Tairāwhiti, N Island NZ | M | 4837 | 15.00 (16.10) 16.88 | 0.5 | 9.69 (9.94) 10.31 | 0.2 |
|  |  |  |  | B | 4838 | 14.69 (16.16) 16.88 | 0.56 | 9.69 (10.00) 10.31 | 0.21 |
| 3 | *L. scoparium* s.l. | 20131121-01 LN | Willi Kaa apiary site, Tikitiki, Tairāwhiti, N Island NZ | M | 4839 | 13.44 (15.65) 17.19 | 0.83 | 9.38 (10.03) 11.25 | 0.71 |
|  |  |  |  | B | 4840 | 13.44 (15.98) 19.06 | 1.39 | 8.44 (9.84) 10.63 | 0.74 |
| 4 | *K. robusta* | 20131125-01 LN | Willi Evans apiary site, Te Araroa, Tairāwhitii, N Island NZ | M | 4841 | 12.81 (13.50) 15.00 | 0.53 | 7.81 (8.59) 9.38 | 0.45 |
|  |  |  |  | B | 4842 | 11.25 (13.55) 15.63 | 0.79 | 8.44 (8.84) 9.38 | 0.3 |
| 5 | *K. robusta* | 20131125-02 LN | Willi Evans apiary site, Te Araroa, Tairāwhitii, N Island NZ | M | 4843 | 12.19 (13.14) 14.06 | 0.49 | 7.50 (7.97) 8.75 | 0.34 |
|  |  |  |  | B | 4844 | 14.06 (13.32) 18.44 | 0.55 | 10.63 (8.34) 11.25 | 0.42 |
| 6 | *K. robusta* | 20131125-06 LN | Willi Evans apiary site, Te Araroa, Tairāwhitii, N Island NZ | M | 4845 | 11.88 (12.98) 15.00 | 0.57 | 8.75 (9.38) 10.00 | 0.39 |
|  |  |  |  | B | 4846 | 11.88 (13.18) 14.38 | 0.58 | 8.13 (8.88) 10.00 | 0.56 |

^a^M = male flowers, B = bisexual flowers.

^b^E and P and standard deviations are in µm.

**Table 2. Pooled mean dimensions for the three male and bisexual flower specimens of each species.**

| S**pecies** | **Flower sex** | **E (µm), n = 90** | **P (µm), n = 30** |
| --- | --- | --- | --- |
| *Leptospermum scoparium* s.l. | male | 16.27 ± 0.95 | 10.27 ± 0.59 |
|  | bisexual | 16.42 ± 1.07 | 10.23 ± 0.62 |
| *Kunzea robusta* | male | 13.20 ± 0.56 | 8.65 ± 0.69 |
|  | bisexual | 13.35 ± 0.66 | 8.69 ± 0.48 |

There appears to be only a very slight difference in equatorial dimension between pollen of male and bisexual flowers in individual plants of each species, the bisexual flowers having fractionally larger pollen: E up to 0.33 µm greater in *L. scoparium*, and up to 0.20 µm greater in *K. robusta*. However, calculation of the standard error of the difference between the means of E for the pooled results from male and bisexual flowers of each species shows that the differences are not significant at the 5% probability level.

Since there appears to be no significant difference between dimensions of pollen of male and bisexual flowers, the individual specimen results for both types of flowers in each species were pooled.

**Table 3. Pooled mean dimensions for each species.**

| S**pecies** | **E (µm), n = 180** | **P (µm), n = 60** |
| --- | --- | --- |
| *Leptospermum scoparium* s.l. | 13.44 – 19.38; 16.35 ± 1.39 | 8.44 – 11.25; 10.25 ± 0.84 |
| *Kunzea robusta* | 11.25 – 18.44; 13.28 ± 0.62 | 7.50 – 11.25; 8.67 ± 0.59 |

**Conclusion**

From these measurements, *Leptospermum scoparium* s.l. pollen equatorial and polar dimensions were seen to be significantly larger than those of the sampled *Kunzea robusta* population, but there is overlap in size between the larger specimens of *K. robusta* and smaller specimens of *L. scoparium*. The overlap in equatorial size is due to a small proportion of specimens: there is no overlap between the one standard deviation ranges (14.96 –17.73 µm and 12.66 – 13.89 µm respectively), and only a 0.94 µm overlap between the two standard deviation ranges (13.57 – 19.12 µm and 12.04 – 14.51 µm).
