# Supplementary figures and images for: Discrimination of pollen of New Zealand mānuka (Leptospermum scoparium agg.) and kānuka (Kunzea spp.) (Myrtaceae)
Source: PLoS One. 2022 Jun 3;17(6):e0269361. doi: 10.1371/journal.pone.0269361 (PMC9165797; doi:10.1371/journal.pone.0269361)

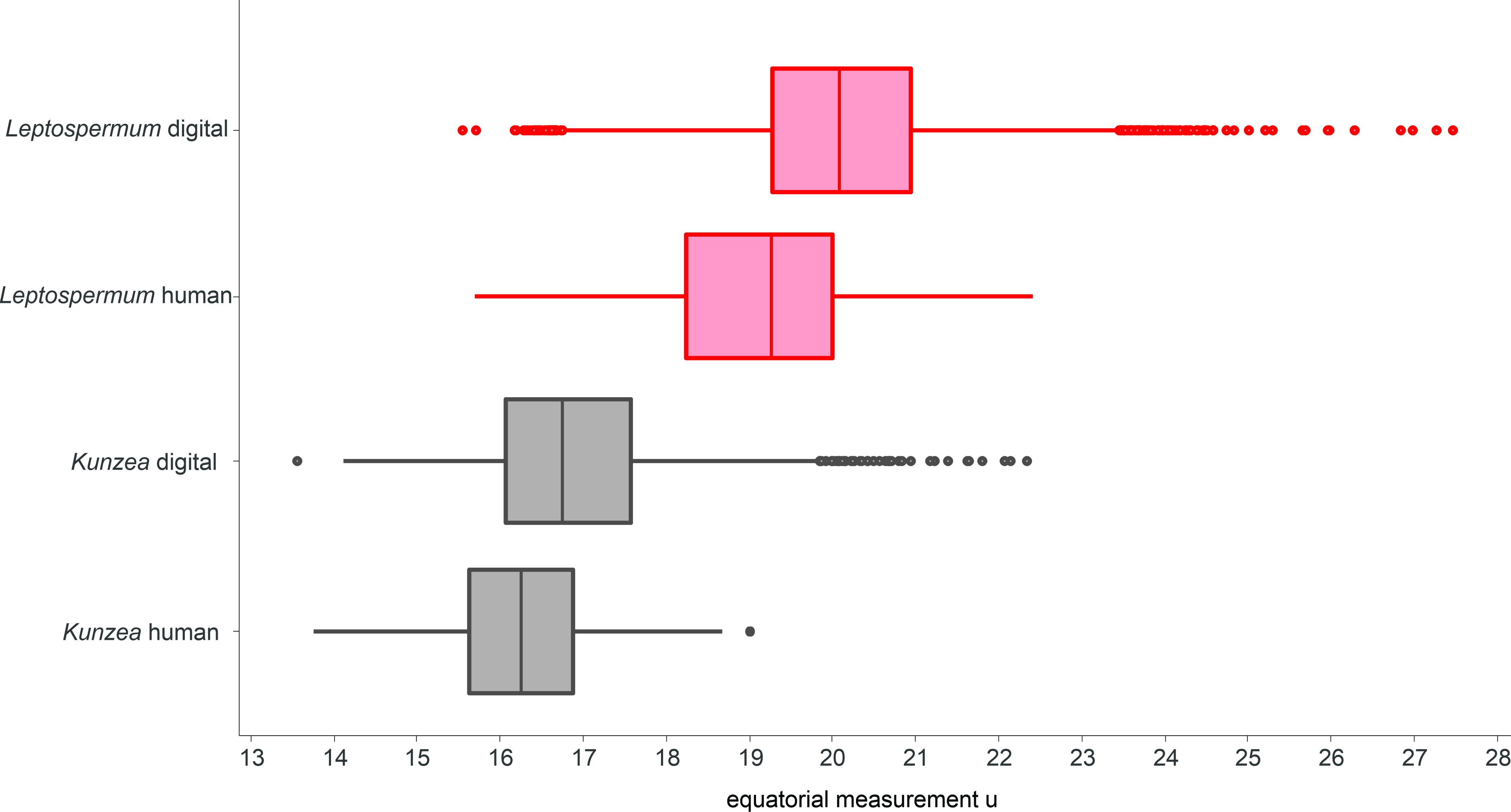

Supplement: S1 Fig — Comparison for Leptospermum scoparium (red) and Kunzea (grey) of equatorial diameter measured by palynologist using a light microscope, and maximum Feret diameter measured by Classifynder. (TIF) [file pone.0269361.s002.tif]

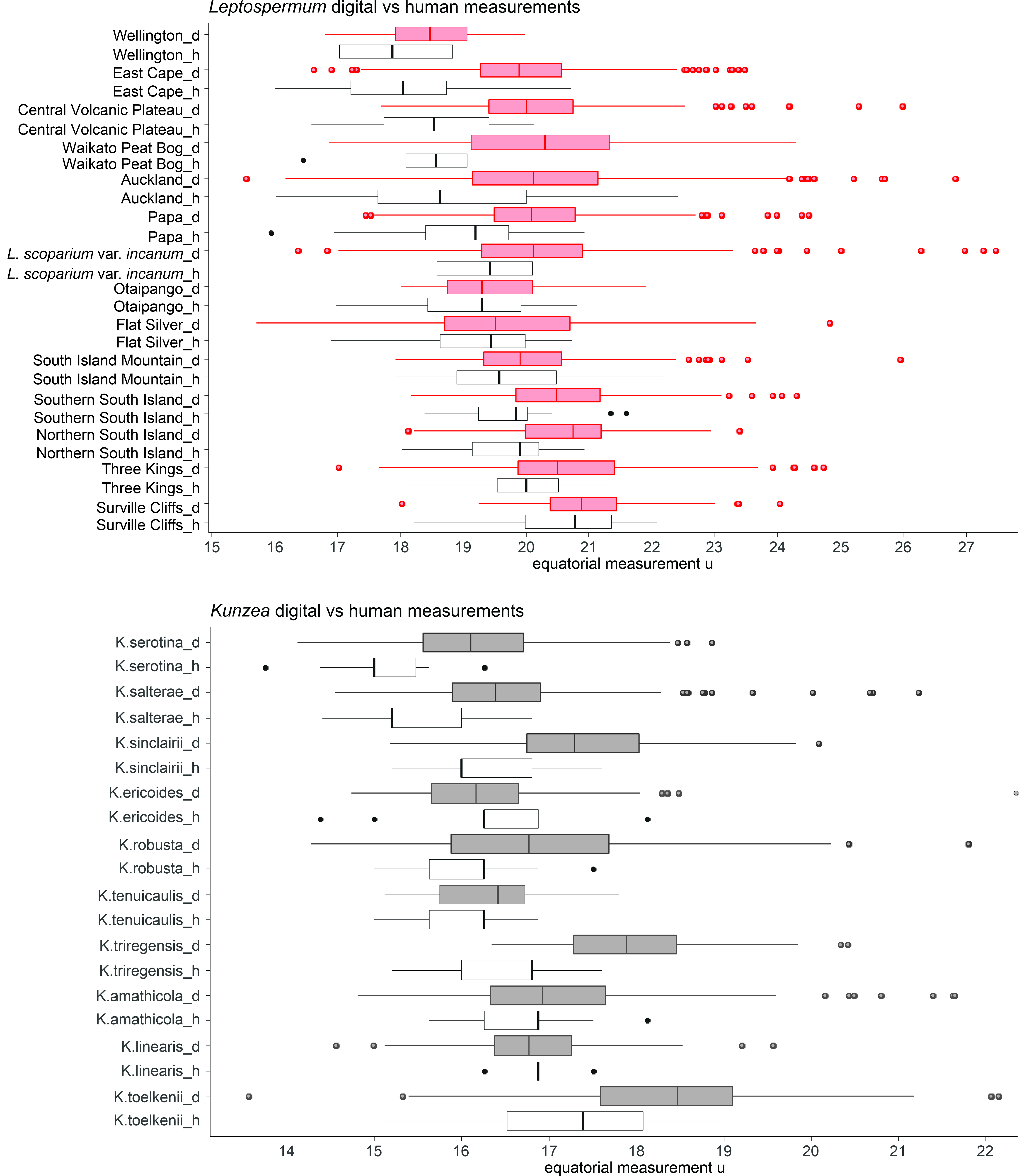

Supplement: S2 Fig — Comparison for Leptospermum scoparium (upper) and Kunzea (lower) of equatorial diameter measured by palynologist using a light microscope (denoted by suffix “_h”), and maximum Feret diameter measured by Classifynder (denoted by suffix “_d”). (TIF) [file pone.0269361.s003.tif]
